# Peripheral Enthesitis Detected by Ultrasonography in Patients With Axial Spondyloarthritis—Anatomical Distribution, Morphology, and Response to Tumor Necrosis Factor-Inhibitor Therapy

**DOI:** 10.3389/fmed.2020.00341

**Published:** 2020-07-15

**Authors:** Sengul Seven, Susanne Juhl Pedersen, Mikkel Østergaard, Sara Kamp Felbo, Inge Juul Sørensen, Uffe Møller Døhn, Lene Terslev

**Affiliations:** ^1^Copenhagen Center for Arthritis Research and Center for Rheumatology and Spine Diseases, Centre of Head and Orthopaedics, Rigshospitalet, Glostrup, Denmark; ^2^Department of Clinical Medicine, University of Copenhagen, Copenhagen, Denmark

**Keywords:** imaging, ultrasound, enthesitis, spondyloarthritis, inflammation

## Abstract

**Objectives:** To investigate the anatomical distribution, morphological abnormalities and response to adalimumab therapy of ultrasound(US)-detected peripheral enthesitis in patients with axial spondyloarthritis (SpA).

**Methods:** In a randomized, placebo-controlled, double-blinded, investigator-initiated trial (NCT01029847), patients with axial SpA according to the Assessment of Spondyloarthritis International Society criteria were randomized to subcutaneous adalimumab 40 mg every other week or placebo from baseline to week 6. From week 6 to 24, all patients received adalimumab 40 mg every other week. Of 49 patients enrolled, 21 patients participated in our observational US sub-study. US assessment applying the OMERACT US definitions for enthesitis of 10 peripheral entheseal regions of the upper and lower extremities and clinical examination were performed at baseline, weeks 6 and 24. US was performed by one experienced investigator. Hypo-echogenicity, increased thickness and Doppler activity of the enthesis were considered signs of active inflammation, whereas insertional bone erosions, intratendinous calcifications, and enthesophytes were regarded as signs of structural lesions.

**Results:** Enthesitis on US was mostly present in the lower limbs, especially in the Achilles tendon (81%), the quadriceps tendon (62%), and the greater femoral trochanter (52%). Structural lesions were predominant (38 vs. 12% of examined entheses with inflammatory changes), particularly in the entheses of the lower limbs, and exhibited no change during treatment.

**Conclusion:** US-detected structural lesions were common while inflammatory lesions were relatively rare in patients initiating adalimumab due to axial SpA. Structural lesions did not appear to change during 24 weeks follow-up, suggesting that these lesions may not be helpful outcome measures in short-term clinical trials.

## Introduction

Enthesitis is typically defined as inflammation of the insertion of tendons, ligaments, aponeurosis, and capsules into the bone, and it is considered a pathological, clinical, and imaging hallmark of the spondyloarthritis (SpA) group, including psoriatic arthritis (PsA) (1–3). The Assessments in the SpondyloArthritis International Working Group (ASAS) and the Group for Research and Assessment of Psoriasis and Psoriatic Arthritis (GRAPPA) have recommended enthesitis as one of the key domains for assessing disease activity and response in SpA (axial and peripheral) and PsA (4, 5). The Outcome Measures in Rheumatology (OMERACT) Ultrasound (US) Working Group (WG), has developed and validated consensus-based US definitions for enthesitis lesions in SpA including PsA (1, 2) of which some are related to inflammation and some to inactive structural lesions.

Enthesitis lesions may be detected by US at clinically asymptomatic entheses and with a greater sensitivity than clinical examination (3–5). Since 1994, US has been used for evaluating peripheral enthesitis in SpA patients in both lower and upper limb entheses (6–9). B-mode and Doppler US (color and power) both depict the morphological features and vascularity of the enthesis and may aid in the diagnosis and evaluation of treatment effect (10–12), however, in most studies the inclusion criterion was symptomatic entheses in addition to US verified Doppler activity in the entheses. Different clinical enthesitis scores (13–15) and US enthesitis scores (5, 16, 17) exist in literature, but currently there is no consensus on which clinical scores and US scores to apply. Additionally, little is known about the presence and response to treatment of US-detected enthesitis (inflammatory lesions and/or structural lesions) in axial SpA patients initiating TNF-I therapy due to axial inflammatory activity.

The aim of the study was to investigate the anatomical distribution, morphological abnormalities and response to Tumor Necrosis Factor-inhibitor (TNF-I) therapy of US-detected peripheral enthesitis lesions in a cohort of patients with axial SpA, with or without symptomatic peripheral enthesitis, initiating adalimumab therapy, applying the OMERACT US definitions for enthesitis lesions.

## Patients and Methods

### Study Design

The main study (the ASIM study) was a randomized, double-blinded, placebo-controlled investigator-initiated, 52 weeks longitudinal trial (ClinicalTrials.gov, NCT01029847) conducted in Denmark at five rheumatology outpatient clinics from 2010 to 2014. Fifty patients were included and randomized to receive subcutaneous adalimumab 40 mg every other week or placebo from baseline to week 6. From week 6 to 24, all patients received adalimumab 40 mg every other week. Participants in our observational US sub-study were recruited among patients in the main study. The US sub-study was conducted at Rigshospitalet, Glostrup. The study was approved by the local ethical committee, approval number H1-2013-118, and conducted according to the Declaration of Helsinki V and the Danish legislation. All participants gave written informed consent before study inclusion.

### Inclusion and Exclusion Criteria

All patients had axial SpA according to the Assessment of Spondyloarthritis International Society (ASAS) classification criteria, sacroiliitis on X-ray or MRI, disease activity assessed by BASDAI >4 (0–10) despite NSAID treatment and a clinical indication for TNF-I treatment. Treatment with glucocorticoids and/or initiation or changes in csDMARD were not allowed 4 weeks prior to inclusion. Entheseal involvement was not an inclusion criterion.

### Patient Evaluation

Patient demographics, clinical and biochemical data were obtained for all participants at every visit. The clinical examination included 66/68 joint count, the Bath Ankylosing Spondylitis Metrology Index (BASMI) and assessment of entheses according to the Leeds enthesitis index (LEI) (13), Maastricht Ankylosing Spondylitis Enthesitis Score (MASES) (15) and the Spondyloarthritis Research Consortium of Canada (SPARCC) enthesitis index (14). A standardized approach to clinical examination of entheses based on a predefined illustrated set of instructions was developed (18). Blood samples were analyzed for serum C-reactive protein (CRP) and Human Leukocyte Antigen B27 (HLA-B27). US assessments were performed at baseline, and weeks 6 and 24.

### Ultrasound

All US scans were performed with General Electric Logiq 9 US machine. A ML 12 high-frequency linear probe was used with a center frequency of 14 megahertz (MHz). Doppler setting was adjusted for slow flow according to published recommendations (19). US was performed blinded to clinical and biochemical data according to a standardized protocol by an experienced investigator (LT), who has previously participated in reliability exercises on patients with enthesitis showing high inter- and intrareader reliability (2). Twenty entheseal sites were examined by greyscale and color Doppler; the common extensor, and flexor tendons of the elbow, insertions of supraspinatus tendon, triceps, greater femoral trochanter, quadriceps, proximal, and distal patellar and Achilles tendon, and plantar fascia. Enthesitis was defined according to the OMERACT definitions (2). Hypo-echogenicity, increased thickness (morphologic abnormalities) and Doppler activity of the enthesis were considered signs of active inflammation, whereas insertional bone erosions, intra-tendinous calcifications, and enthesophytes were regarded as signs of structural lesions (2). All lesions were scored dichotomously. US enthesitis was scored according to the Glasgow Ultrasound Enthesitis Scoring System (GUESS) (5), Spanish Enthesitis Index (SEI) (16) and the Madrid Sonography Enthesitis Index (MASEI) (17).

## Statistics

Descriptive statistics were applied for analysis of demographics, clinical, biochemical, and US data. Statistical analyses were performed using non-parametric tests. Differences in the two study populations were analyzed by applying Chi^2^-test and Mann-Whitney *U*-test, and the Wilcoxon Rank test was used to analyze differences in treatment effect over time. To determine the agreement between clinical enthesitis vs. US enthesitis and US inflammation, respectively, Cohen's Kappa was calculated, and Spearman's Rho was performed to calculate correlations between clinical scores vs. US enthesitis scores at baseline. We considered Kappa values of <0 as indicating no agreement, 0–0.20 as slight, 0.21–0.40 fair, 0.41–0.60 moderate, 0.61–0.80 good, and 0.81–1 as excellent agreement. A *p* < 0.05 was considered statistically significant. All statistical analyses were performed in IBM SPSS version 22.0.

## Results

### Demographics, Clinical, and Biochemical Characteristics

Of the 49 patients enrolled in the main study, 21 participated in the US sub-study. The number of patients randomized to receive adalimumab and placebo were 11 and 10, respectively. The patients were 57% males with a mean age of 39.4 years, and 71% were HLA-B27 positive. Baseline demographics, clinical and biochemical characteristics are shown in Table 1. The two randomization groups were comparable in baseline characteristics apart from age where patients in the adalimumab group were statistically significantly older than in the placebo group.

**Table 1:** Demographic, clinical, and laboratory characteristics of the study population.

	**All patients**	**Placebo**	**Adalimumab**	*P*-value^*^
Number of participants	21	10	11	
**Demographics**				
Age, years	39.4 (9.4)	34.3 (5.5)	44.1 (10.0)	**0.02**
Female sex, *N* (%)	9 (42.9)	5 (50.0)	4 (36.4)	0.5
Symptom duration, years	14.1 (12.8)	11.1 (6.8)	16.8 (16.4)	0.9
**Biochemical characteristics**				
HLA-B27 positive, *N* (%)	15 (71.4)	8 (80.0)	7 (63.6)	0.4
C-reactive protein (mg/L)	8.5 (15.9)	3.8 (3.5)	12.8 (21.3)	0.3
**Clinical characteristics**				
Physician VAS global (0–10)^a^	6.8 (2.0)	7.7 (1.4)	5.9 (2.2)	0.2
SJC66	0.2 (0.6)	0.2 (0.6)	0.3 (0.7)	0.6
TJC68	3.6 (7.0)	3.6 (4.3)	3.6 (9.0)	0.4
MASES (0–13)	3.9 (3.8)	4.6 (4.0)	2.9 (3.7)	0.3
LEI (0–6)	0.9 (1.0)	0.7 (1.0)	1.1 (1.1)	0.5
SPARCC enthesitis index (0–16)	3.0 (3.2)	3.2 (3.8)	2.6 (2.9)	1.0
**Patient reported outcomes**				
HAQ	1.0 (0.5)	1.1 (0.7)	1.0 (0.4)	0.6
VAS pain (0–10)^a^	7.3 (2.0)	7.7 (1.8)	6.9 (2.1)	0.4
VAS patient global (0–10)^a^	7.2 (2.1)	7.6 (2.2)	6.8 (2.1)	0.3
BASDAI (0–10)	6.6 (1.6)	7.0 (1.9)	6.3 (1.3)	0.4
BASFI (0–10)	5.9 (2.1)	5.9 (2.5)	5.8 (1.9)	0.9
BASMI (0–10)	3.2 (2.1)	2.7 (2.0)	3.7 (2.2)	0.2

N (%) for female sex and HLA-B27 status, mean (SD) for all others. Chi^2^-test for female gender and HLA-B27, Mann-Whitney U-tests for all others.

* P < 0.05 is considered significant and indicated in bold.

a According to Bath Ankylosing Spondylitis Questionnaire.

BASDAI, Bath Ankylosing Spondylitis Disease Activity Index; BASFI, Bath Ankylosing Spondylitis Functional Index; BASMI, Bath Ankylosing Spondylitis Metrology Index; HAQ, Health Assessment Questionnaire; HLA-B27, Human Leukocyte Antigen B27; LEI, Leeds Enthesitis Index; MASES, Maastricht Ankylosing Spondylitis Enthesitis Score; SJC, Swollen Joint Count; SPARCC, Spondyloarthritis Research Consortium of Canada; TJC, Tender Joint Count; VAS, Visual Analog Scale.

### The Distribution of US Enthesitis at Baseline

The distribution of clinical and US signs of enthesitis in all 21 patients at baseline is provided in Table 2, including the Kappa agreement between clinical enthesitis vs. US enthesitis (i.e., inflammatory lesions and/or structural lesions) and clinical enthesitis vs. US inflammatory lesions alone. Overall, enthesitis (inflammatory lesions and/or structural lesions) was found in 95% of patients. Inflammatory lesions were found in 52% of patients (12% of examined entheses—2% with Doppler activity), while 95% of patients (38% of examined entheses) had structural lesions perceived to be inactive. In comparison 67% of patients had clinical signs of enthesitis. The US-inflammatory lesions in the lower extremities were most frequently found in the insertions of the Achilles tendon (19%) and plantar fascia (19%), while in the upper extremities in the insertion of triceps tendon (5%) and the common extensor tendon of the elbow (5%). Inflammatory lesions were not seen at the greater femoral trochanter and at the insertion of the common flexor tendon.

**Table 2:** Distribution of clinical and US entheseal findings and enthesitis scores at baseline (*n* = 21).

**Entheseal region**	**US**			**Clinical enthesitis^**c**^**
	**Inflammatory lesions^**a**^**	**Structural lesions^**b**^**	**Enthesitis (inflammatory and/or structural lesions)**	
Supraspinatus tendon	0	6 (28.6)	6 (28.6)^##^	8 (38.1)
Triceps tendon	1 (4.8)	1 (4.8)	2 (9.5)	NA
Common extensor tendon, elbow	1 (4.8)^$^	5 (23.8)	5 (23.8)^#^	3 (14.3)
Common flexor tendon, elbow	0	0	0	6 (28.6)
Greater femoral trochanter	0	11 (52.4)	11 (52.4)^##^	12 (57.1)
Quadriceps tendon	3 (14.3)^$^	13 (61.9)	13 (61.9)^#^	3 (14.3)
Proximal insertion of the patellar tendon	1 (4.8)^$^	2 (9.5)	3 (14.3)^#^	4 (19.0)
Distal insertion of the patellar tendon	2 (9.5)^$^	2 (9.5)	3 (14.3)^#^	2 (9.5)
Achilles tendon	4 (19.0)^$$$^	16 (76.2)	17 (81.0)^#^	4 (19.0)
Plantar fascia	4 (19.0)^$^	0	4 (19.0)^#^	6 (28.6)
**US enthesitis score**				
GUESS (0–36)		3.1 (1.9)		
SEI (0–76)		1.8 (2.1)		
MASEI (0–136)		6.7 (4.6)		

N (%) participants with lesions.

a Inflammation: Doppler activity, hypo-echogenicity or increased thickness.

b Chronic lesions: Erosions, calcifications or enthesophytes.

c Entheses with tenderness.

Kappa agreement was calculated for US enthesitis vs. clinical enthesitis. Kappa value <0 no

#, 0–0.20 slight

##, 0.21–0.40 fair, 0.41–0.60 moderate, 0.61–0.80 good, 0.81–1 excellent agreement, respectively.

Kappa agreement was calculated for US inflammatory lesion vs. clinical enthesitis. Kappa value <0 no

$, 0–0.20 slight

$$, 0.21–0.40 fair

$$$, 0.41–0.60 moderate, 0.61–0.80 good, 0.81–1 excellent agreement, respectively.

*GUESS, The Glascow Ultrasound Enthesitis Scoring System; LEI, Leeds Enthesitis Index; MASEI, Madrid Sonography Enthesitis Index; MASES, Maastricht Ankylosing Spondylitis Enthesitis Score; NA, not applicable; SEI, Spanish Enthesitis Index; SPARCC, Spondyloarthritis Research Consortium of Canada; US, ultrasound*.

US enthesitis (inflammation and/or structural US changes) was predominantly found in the lower extremities, especially in the Achilles tendon (81%), the quadriceps tendon (62%) and at the insertion onto the greater femoral trochanter (52%), and these were mostly structural lesions (76, 62, 52%, respectively). In the upper extremities US enthesitis was mostly recorded in the supraspinatus tendon insertion (29%) and at the insertion of the common extensor tendon of the elbow (24%).

When evaluating the different types of inflammatory and structural lesions (Table 3), at the upper extremities we observed the presence of erosion only at the insertion of the supraspinatus tendon (18%) and the common extensor tendon of the elbow (5%), while erosions were recorded at nearly all entheses of the lower extremities, especially at the greater femoral trochanter (33%), although never in the distal insertion of the patellar tendon. Calcifications and/or enthesophytes were present across all entheseal regions, except for the common flexor tendon of the elbow and the plantar fascia. Increased thickness and/or hypoechoic features were mostly seen at the Achilles tendon insertion and the plantar fascia (14 and 15%, respectively), while completely absent at the supraspinatus tendon, common flexor tendon of the elbow, and the greater femoral trochanter. Doppler was only recorded at the quadriceps tendon (7%) and the distal insertion of the patellar tendon (2%).

**Table 3:** US findings at baseline (*n* = 21).

**Entheseal region**	**Erosion**	**Calcifications / enthesophytes**	**Increased thickness / hypoechoic**	**Doppler**
Supraspinatus tendon	7 (18)	2 (5)	0	0
Triceps tendon	0	1 (3)	1 (2)	0
Common extensor tendon, elbow	2 (5)	6 (14)	2 (5)	0
Common flexor tendon, elbow	0	0	0	0
Greater femoral trochanter	14 (33)	6 (14)	0	0
Quadriceps tendon	3 (7)	20 (48)	2 (5)	3 (7)
Proximal insertion of the patellar tendon	3 (7)	2 (5)	1 (2)	0
Distal insertion of the patellar tendon	0	3 (7)	1 (2)	1 (2)
Achilles tendon	3 (7)	28 (67)	6 (14)	0
Plantar fascia (*n* = 20)	1 (3)	0	6 (15)	0

*N (%) of entheses with lesions*.

### Agreement Between US Enthesitis and Clinical Assessment

A fair agreement for US inflammatory lesions vs. clinical enthesitis was seen at the Achilles tendon insertion, while none to poor agreement was found between US structural lesions and inflammatory lesions vs. clinical enthesitis for all other entheses (Table 2). Overall, 10% of non-tender entheses showed US signs of inflammation and on the contrary 18% of tender entheses did not show US signs of inflammation. The different clinical scores of enthesitis and the different US enthesitis scores at baseline are also seen in Table 2. When performing the Spearmann's rho, we only found a statistically significant correlation between the LEI clinical enthesitis score vs. SEI US enthesitis score.

### US and Clinical Enthesitis Changes During Treatment

The clinical findings and US enthesitis (inflammatory and/or structural lesions) scores in the two randomization groups at weeks 0, 6, and 24 are provided in Table 4. A statistically significant decrease in BASDAI was seen at both weeks 6 and 24 in the adalimumab group, while only at week 24 in in the placebo group. The SPARCC enthesitis index had decreased significantly from baseline at weeks 6 and 24. No other changes in clinical indices was observed in either of the groups. Regarding the US findings, the only statistically significant changes were for the common extensor tendon of the elbow from baseline to weeks 6 and 24 in the adalimumab group, and for the SEI US score from baseline to week 24 in the placebo group.

**Table 4:** Clinical and US findings during study period.

	**Placebo**			**Adalimumab**		
	**Week 0**	**Week 6**	**Week 24**	**Week 0**	**Week 6**	**Week 24**
Number of patients	10	9	10	11	11	10
**Clinical enthesitis scores**						
MASES (0–13)	4.6 (4.0)	4.0 (3.3)	2.2 (3.7)	3.0 (3.7)	2.1 (2.5)	2.0 (3.0)
LEI (0–6)	0.7 (0.9)	0.9 (1.0)	0.6 (0.9)	1.1 (1.1)	0.7 (0.9)	0.3 (0.7)
SPARCC enthesitis index (0–16)	3.2 (3.8)	3.6 (2.6)	2.2 (2.8)	2.6 (2.9)	1.6 (1.9)^**^	0.8 (1.1)^***^
BASDAI	7.0 (1.9)	6.4 (2.5)	4.3 (2.7)^***^	6.3 (1.3)^*^	4.2 (2.3)^**^	2.9 (2.5)^***^
**US findings**^**a**^						
Supraspinatus tendon	3 (30)	2 (22)	1 (10)	3 (27)	2 (18)	1 (10)
Common extensor tendon, elbow	0	0	0	5 (46)^*^	0	1 (10)^***^
Common flexor tendon, elbow	0	0	0	0	0	0
Triceps tendon, elbow	1 (10)	0	1 (10)	1 (9)	0	2 (20)
Greater femoral trochanter	6 (60)	6 (67)	6 (60)	5 (46)	6 (55)	6 (60)
Quadriceps tendon	4 (40)	1 (11)	4 (40)	9 (82)	8 (73)	6 (60)
Proximal insertion of the patellar tendon	1 (10)	0	0	2 (18)	1 (9)	1 (10)
Distal insertion of the patellar tendon	2 (20)	1 (11)	1 (10)	1 (9)	1 (9)	0
Achilles tendon	7 (70)	7 (78)	8 (80)	10 (91)	7 (64)	7 (70)
Plantar fascia	1 (10)	0	0	3 (27)	3 (27)	2 (20)
**US enthesitis scores**						
GUESS (0–36)	2.90 (2.33)	2.56 (1.59)	2.22 (1.39)	3.18 (1.60)	3.09 (1.92)	2.56 (1.81)
SEI (0–76)	2.10 (2.13)	1.22 (1.30)	0.89 (1.54)^***^	1.55 (2.16)	1.18 (0.98)	0.89 (1.05)
MASEI (0–136)	6.00 (4.92)	4.89 (3.06)	5.00 (3.87)	7.36 (4.46)	7.36 (4.59)	6.89 (5.13)

Mean (SD) for clinical and US enthesitis scores, n (%) participants with US findings.

a Inflammatory and/or structural lesions.

Wilcoxon Rank test was applied. P < 0.05 is considered significant.

* p < 0.05 week 0–6;

** P < 0.05 week 6–24;

*** P < 0.05 week 0–24.

*BASDAI, Bath Ankylosing Spondylitis Disease Activity Index; GUESS, The Glascow Ultrasound Enthesitis Scoring System; LEI, Leeds Enthesitis Index; MASEI, Madrid Sonography Enthesitis Index; MASES, Maastricht Ankylosing Spondylitis Enthesitis Score; SEI, Spanish Enthesitis Index; SPARCC, Spondyloarthritis Research Consortium of Canada; US, Ultrasound*.

## Discussion

In this observational US sub-study of axial SpA patients who initiated TNF-I treatment based on axial inflammation, a high prevalence of structural lesions was observed in peripheral entheses (95% of patients, 38% of examined entheses), whereas the prevalence of inflammatory entheseal changes was fairly low (52% of patients, 12% of examined entheses, and 2% with Doppler activity). US signs of enthesitis were mainly identified in the lower extremities, mostly as structural lesions. No change in structural lesions were found during treatment, indicating a low ability to change and supporting the perception of being inactive lesions.

The high frequency of US structural lesions in our population with long-standing disease is in line with previous US studies of enthesitis in SpA patients by Naredo et al. (3) and D'Agostino et al. (20) and in AS patients by Wink et al. (6) and Spadaro et al. (7) Calcifications/enthesophytes can also be seen in healthy subjects (8–11), with the highest frequency in the lower limbs, possibly increasing with increasing age, but with a lower prevalence than in SpA patients.

Regarding US signs of entheseal inflammation, the study by Wink et al. (6), which included AS patients, found Doppler activity in 55% of examined entheses, (i.e., more frequently compared with our study). Wink et al. did not include the insertions of triceps or supraspinatus but included the pes anserine. The latter may explain the high frequency of Doppler findings, as this was the most frequent site for Doppler activity. The proximity of the pes anserine to the inferior geniculate artery might increase the risk of overestimation of the inflammation. Since this study also looked at combinations of inflammatory lesions and included adjacent bursitis and effusion in this definition, results are therefore not comparable. Spadaro et al. (7) found a frequency of entheseal Doppler activity (6%) more similar to ours. However, Naredo et al. (20) also found a high prevalence of inflammatory changes with morphologic abnormalities in 61% of the SpA patients and intra-entheseal Doppler activity in 47% of the patients, both most commonly at the Achilles tendon insertion (29 and 16%, respectively). Our population, however, was selected based on axial manifestations, while peripheral findings were not required. This explains the low frequency of inflammatory US findings. The frequently registered high frequency of structural changes in peripheral entheses documents that most patients with axial SpA at some stage in their disease course get entheseal affection which is severe enough to leave recognizable structural damage.

Entheseal Doppler activity has been found in healthy subjects (8, 12, 21), also at the quadriceps insertion, where most Doppler activity was also found in our population. Other studies did not find any entheseal Doppler activity in healthy subjects (9, 22).

Although not an inclusion criterion, the patients in our study had clinically peripheral enthesis involvement with a MASES index of 3.9 (3.8), however, there were none to poor agreement between clinical entheses assessment and US findings of enthesitis. Further, no agreement was seen when evaluating different clinical enthesis scores and different US enthesitis scores. This is in line with several previous studies that have found US to be more sensitive than clinical evaluation for detection of inflammation (4). The tenderness at the clinically tender entheses that did not show US signs of inflammation may originate from other tender structures close to the entheses.

An outcome measure needs to possess ability to change during effective therapies (23). Wink et al. found no statistically significant decrease in inflammatory lesions (6). In the present study US enthesitis was not sensitive to change during adalimumab therapy, which is probably at least partially explained by the low number of inflammatory lesions. The lack of documented responsiveness supports that future studies should both test the current US measures in more actively inflamed cohorts and investigate new and potentially more sensitive measures.

The strength of this study is the placebo-controlled study design applying a standardized clinical and US protocol performed by an experienced sonographer. However, the study was observational and included axial SpA patients regardless of the presence of US enthesitis findings. Additionally, the low number of patients and low number of inflammatory lesions are the primary limitation, since the statistical power to show significant changes over time and differences between the groups was low.

In conclusion, we found by US a high prevalence of structural lesions and a very low prevalence of inflammatory lesions in a population of axial SpA patients with or without clinical peripheral enthesitis. In our study US structural lesions did not appear to have ability to change during 24 weeks of TNF-I treatment, suggesting that US-structural enthesitis lesions may not be helpful outcome measures in short-term clinical trials.

## Data Availability Statement

The raw data supporting the conclusions of this article will be made available by the authors, without undue reservation.

## Ethics Statement

The studies involving human participants were reviewed and approved by the Videnskabsetiske Komiteer in the Capital Region of Denmark. The patients/participants provided their written informed consent to participate in this study.

## Author Contributions

LT, SP, and MØ contributed to the conception and design of the study. SS, SF, and LT wrote sections of the manuscript. SS and LT wrote the first draft of the manuscript. SS, LT, SF, and SP performed the statistical analysis and interpretation. SS organized the database. LT, IS, SP, and UD contributed to the acquisition of data. All authors contributed to manuscript revision, read, and approved the submitted version.

## Conflict of Interest

SS: research support from Novartis, Honoraria from Sanofi, UCB, and Abbvie. SP: speakers fee from MSD, Pfizer, AbbVie, Novartis, and UCB. Advisory board member for AbbVie and Novartis, research support from AbbVie, MSD, and Novartis. MØ: research support, consultancy fees and/or speaker fees form Abbvie, BMS, Boehringer-Ingelheim, Celgene, Eli-Lilly, Hospira, Janssen, Merck, Novartis, Novo, Orion, Pfizer, Regeneron, Roche, Sandoz, Sanofi, and UCB. SF: research support from Celgene. UD: speaker and consulting fees from Eli-Lilly, Pfizer, Novartis, Roche, and Abbvie. LT: speakers fee from AbbVie, Janssen, Roche, Novartis, Pfizer, MSD, BMS, and GE. The remaining author declares that the research was conducted in the absence of any commercial or financial relationships that could be construed as a potential conflict of interest.
